# Caudate Functional Connectivity Associated With Weight Change in Adolescents

**DOI:** 10.3389/fnhum.2020.587763

**Published:** 2020-11-16

**Authors:** Yuko Nakamura, Sachiyo Ozawa, Shinsuke Koike

**Affiliations:** ^1^UTokyo Center for Integrative Science of Human Behavior, The University of Tokyo, Tokyo, Japan; ^2^International Research Center for Neurointelligence, Tokyo, Japan; ^3^University of Tokyo Institute for Diversity and Adaptation of Human Mind, Tokyo, Japan; ^4^Center for Evolutionary Cognitive Sciences, Graduate School of Arts and Sciences, University of Tokyo, Tokyo, Japan

**Keywords:** adolescents, caudate, lateral prefrontal cortex, resting-state functional connectivity, weight management

## Abstract

**Background:**

Childhood obesity has become a global epidemic and the etiology of maladaptive ingestive behavior in children warrants further research. Mounting evidence suggests that the caudate is associated with body weight gain and obesity in adults. In adolescents, however, how caudate-related neural networks are associated with body weight gain is unclear because their central nervous systems are still developing.

**Objectives:**

The current longitudinal resting-state functional magnetic resonance imaging (rs-fMRI) study was conducted to investigate the hypothesis that caudate-related neural networks have a role in weight gain in adolescents.

**Methods:**

The study included 20 healthy adolescents with a mean age of 17.5 ± 2.0 years and a mean body mass index of 20.6 ± 2.1 who underwent baseline rs-fMRI then follow-up rs-fMRI approximately 1 year later. Body mass index (BMI) was measured at both timepoints. Seed-based functional connectivity analysis was utilized to analyze caudate-related functional connectivity (FC) using the caudate as a seed. Associations between caudate-related FC and BMI at baseline were assessed, as were associations between change in BMI and caudate-related FC between baseline and follow-up.

**Results:**

At baseline, greater caudate-lateral prefrontal cortex FC was correlated with lower BMI (family wise error-corrected *p* < 0.05). Compared to the baseline, increased FC in the caudate-lateral prefrontal cortex at follow up were negatively associated with increased BMI (*p* < 0.05).

**Conclusion:**

Given that the lateral prefrontal cortex and caudate are associated with inhibitory control, the caudate-lateral prefrontal cortex FC may have a preventive effect on weight gain in adolescents. The results of the current study suggest that developing inhibitory control would lead to the prevention of childhood obesity.

## Introduction

Childhood obesity has become a global epidemic in the last three decades (Abarca-Gómez et al., 2017), and it may cause deficits in structural and functional brain development (Laurent et al., 2019; Ronan et al., 2020) as well as various non-communicable diseases such as diabetes and cardiovascular conditions (Sahoo et al., 2015). A better understanding of the neuropathology of childhood obesity is thus needed to improve strategies for its prevention and treatment.

Weight change is reportedly associated with structural and functional alterations in reward regions of the brain such as the midbrain, pallidum, orbitofrontal cortex (Stice and Yokum, 2016), and especially the caudate. In one study, reduced caudate responses to palatable liquid consumption were correlated with increased body mass index (BMI), and this caudate response was associated with weight gain at a 1-year follow-up timepoint (Stice et al., 2008a). In another study, reduced caudate responses to sugary liquid consumption were correlated with increased BMI at a 6-month follow-up timepoint in women (Stice et al., 2010). In adolescents, altered caudate responses to food cues were reportedly associated with body fat gain at a 3-year follow-up timepoint (Stice et al., 2015) and BMI gain at an 1-year follow-up (Yokum et al., 2014). In a weight loss trial, greater reduction in brain responses to high-calorie food cues at a 1-month follow-up timepoint predicted greater weight loss at a 6-month follow-up timepoint (Hermann et al., 2019). In addition, altered caudate-related functional connectivity is reportedly predictive of increased BMI 6-months later (Gao et al., 2018) and 1 year later (Contreras-Rodríguez et al., 2017). Collectively, these observations suggest that aberrant functions or connectivity in the caudate are likely to be linked to disrupted weight maintenance.

The caudate is deep inside the brain near the thalamus, and is connected to parts of the brain associated with nearly all of its facets, including cognition, emotion, perception, and action-related structures and networks (Grahn et al., 2008; Robinson et al., 2012). The caudate responses to various food stimuli (van der Laan et al., 2011; Huerta et al., 2014; Arsalidou et al., 2020; Chen and Zeffiro, 2020) and caudate-related networks are changed by internal states (e.g., hunger or fullness) or food consumption in adults (Siep et al., 2009; Contreras-Rodriguez et al., 2019; Chen and Zeffiro, 2020) and adolescents (Page et al., 2013; Jastreboff et al., 2016). The caudate is connected to basal ganglia (e.g., substantia nigra and globus pallidus) as well as the prefrontal cortex, and is involved in reward processing, learning, goal-directed behavior, and decision-making (Balleine et al., 2007). In concert with the prefrontal cortex, it is also involved in inhibitory control (Guo et al., 2018; Cai et al., 2019) and cognitive appetite control (Tuulari et al., 2015). Given that the caudate and related neural networks are associated with perception of food stimuli, reward processing, and cognitive appetite control, it is likely to have an important role in ingestive behavior and weight change.

It has been repeatedly reported that impulsivity is associated with binge eating that could be a cause of excessive weight gain (Meule, 2013) in young adults (Claes et al., 2005; Oliva et al., 2019, 2020) and adolescents (Stice et al., 2013). In addition, increased impulsivity is associated with altered caudate function or connectivity (Babbs et al., 2013; Davis et al., 2013; Oliva et al., 2019) and obstruct function or structure in the prefrontal cortex, which is a key region for inhibitory or self-control behavior (Lowe et al., 2019). Given that adolescents exhibit greater impulsivity than adults because their inhibitory control mechanisms are still developing (Romer, 2010), and they are evidently more prone to over consumption of rewarding high-calorie food (van Meer et al., 2016). It is thus important to elucidate the associations between caudate-related neural networks and body weight change in adolescents, but these associations have not been well characterized. In the current longitudinal study resting-state functional magnetic resonance imaging (rs-fMRI) and seed-based functional connectivity (FC) analysis were used to examine associations between caudate-related FC and BMI change in adolescents. We hypothesized that increased BMI or BMI change would be associated with reduced caudate-related FC in the inhibitory region and increased caudate-related FC in reward processing or motivation regions, which were associated with food reward processing and weight gain (e.g., striatum, midbrain, amygdala, insula, pre-/postcentral gyrus, and orbitofrontal cortex) (García-García et al., 2014; Stice and Yokum, 2016).

## Materials and Methods

### Participants

The study included 20 adolescents (seven male, 13 female) with a mean age of 17.5 years [standard deviation (SD) 2.0, range 14–19 years] and a mean BMI of 20.6 (SD 2.1, range 17.2–25.7) at baseline who were recruited from the metropolitan area of Tokyo. All participants and parents/guardians of the high school or middle school participants provided written informed consent, and the study was approved by the Ethics Committee of the Graduate School of Arts and Sciences at the University of Tokyo (approval number 513-2). All participants were healthy and had no history of psychological or neurological disorders or head injury. Detailed demographic data are shown in Table 1.

**TABLE 1:** Demographics.

	**Baseline**	**1-year follow-up**	**Change**	***p* value**
Sex (male/female)	7/13			
Age (mean ± SD) (range)	17.5 ± 2.0 (14.1–19.9)	18.6 ± 2.0 (15.1–20.8)	1.1 ± 0.3 (0.9–1.8)	<0.001
BMI (mean ± SD) (range)	20.6 ± 2.1 (17.2–25.7)	20.8 ± 1.6 (17.6–23.8)	0.25 ± 2.53 (−4.3 – 6.5)	0.68

*BMI, Body mass index.*

### Experimental Timepoints

All participants underwent rs-fMRI at baseline and approximately 1 year or more thereafter (mean 12.95 ± 3.02 months, range 11–22 months). Within 1 month before or after each rs-fMRI session, each participant visited the laboratory and their weight and height were measured by trained experimenters (YN or SO) using a multi-frequency body composition meter (MC-780A, TANITA CORPORATION, Tokyo, Japan) to calculate BMI (kg/m^2^). We also collected self-reported weight and height on the scanning day. We calculate BMI using self-reported weight and height. There was no significant difference between self-reported BMI and measured BMI (*p* = 0.86, *t* = −0.18 at baseline; *p* = 0.79, *t* = −0.27 at follow-up). Therefore, we assumed that the participants’ BMI did not significantly change within a month from the rs-fMRI scanning day.

At this visit, participants also completed the Japanese version Barratt Impulsiveness Scale Version 11 (BIS-11) (Patton et al., 1995; Someya et al., 2001; Kobashi and Ida, 2013), which was a questionnaire designed to assess the personality/behavioral construct of impulsiveness (Someya et al., 2001). This self-reported questionnaire has been adapted to assess impulsivity in adolescents (Pechorro et al., 2016; Huang et al., 2017). The BIS-11 contains a total of 30 items scored on a Likert scale (ranging from never = 1 point to very frequently = 6 points) yielding impulsivity measures on three scales: attentional (inability to focus or concentrate), motor (tendency to act without thinking), and non-planning impulsivity (lack of future planning and forethought). The total score ranges from 30 to 180, with a higher score indicating greater impulsivity.

### Rs-fMRI Procedure

**FIGURE 1:**
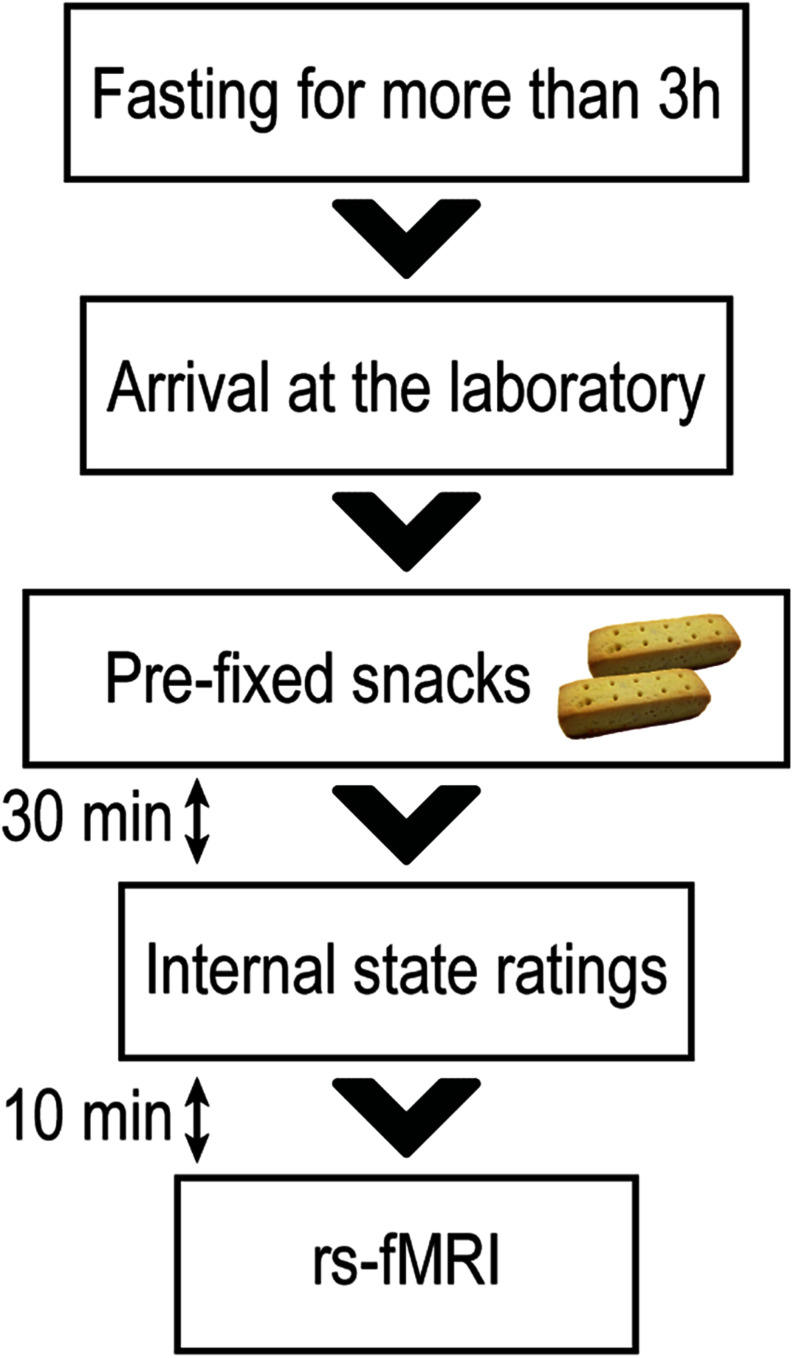
Resting-state functional magnetic resonance imaging session. All participants were instructed to abstain from any food or drinks apart from water for at least 3 h before their visit to the laboratory. Approximately 30 min before the resting-state functional magnetic resonance imaging (rs-fMRI) scanning, each participant was instructed to consume pre-prepared snacks (280 kcal) to standardize their internal states of hunger and fullness. Approximately 10 min before the rs-fMRI scanning, participants rated their levels of hunger and fullness.

Approximately three to 4 h after a meal, the level of appetite generally returns to almost the same level as that of the pre-meal appetite (Leidy et al., 2010; Gibbons et al., 2013; DeBenedictis et al., 2020). Since caudate-related functional connectivity could be changed by internal states (e.g., hunger or fullness) (Siep et al., 2009; Page et al., 2013; Jastreboff et al., 2016; Contreras-Rodriguez et al., 2019; Chen and Zeffiro, 2020), the participants were instructed to abstain from food or drink except for water for at least 3 h before their visit to the laboratory to avoid the effect of the meal on caudate FC. In an effort to standardize participants’ internal states, they were instructed to consume pre-fixed snacks (280 kcal) approximately 30 min before rs-fMRI scanning (Figure 1). The participant was then escorted to the fMRI room to undergo rs-fMRI scanning. Approximately 10 min before rs-fMRI scanning, each participant rated their internal states (e.g., hunger and fullness) using an 8-point Likert scale, where 1 equated to “not at all” and 8 equated to “more than ever.” Before rs-fMRI scanning, each participant underwent an fMRI experiment to measure brain responses to food cues. After the food cue fMRI experiment, we asked all participants if they felt any fatigue or anxiety. All participants responded that they were comfortable to continue scanning. The participant underwent rs-fMRI scanning (10 min 10 s, 244 volumes).

The participant was instructed to relax and lie still in the scanner while remaining calm and awake. During rs-fMRI scanning, the participant was instructed to fix their gaze on a white cross on the black screen, which was projected on a mirror attached to the head-coil from the monitor that was set behind the bore of the MRI machine.

### Image Acquisition

Images were acquired on a 3 Tesla MRI scanner (Magnetom Prisma Fit, Siemens Medical Systems, Munich, Germany) using a 64-channel head/neck coil. T2^∗^-weighted images reflecting blood oxygen level-dependent signals were acquired using gradient-echo echo-planar imaging (repetition time 2500 ms, echo time 30 ms, 38 slices, flip angle 80°, field of view 212 mm × 212 mm, and resolution 3.3 mm × 3.3 mm × 4.0 mm) in ascending order (10 min and 10 s). Anatomical images were acquired using a T1-weighted 3D MPRAGE protocol (repetition time 1900 ms, echo time 2.53 ms, flip angle 9°, field of view 256 mm × 256 mm, resolution 1.0 mm × 1.0 mm × 1.0 mm).

### Rs-fMRI Data Preprocessing

Resting-state functional magnetic resonance imaging data were preprocessed using FSL (version 6.0) (Jenkinson et al., 2012) and AFNI (version 20.0.05 “Galba”) (Cox, 1996) software. The first four volumes of each functional time series were excluded from the analysis to allow for magnetization equilibrium. Conventional preprocessing was conducted as follows: (1) Head-motion correction via realignment of the time-series to the middle volume; (2) fieldmap-based distortion-correction; (3) slice-timing correction; (4) non-brain tissue removal using the brain extraction tool; (5) spatial smoothing with a 5-mm full-width at half maximum Gaussian kernel; (6) high-pass temporal filtering (1/150 Hz cutoff); and (7) linear detrending. The exclusion criterion for head-motion was greater than one voxel size (3.3 mm × 3.3 mm × 4.0 mm). No participant met this exclusion criterion, thus data from all participants were included in further rs-fMRI analyses in which head-motion in rs-fMRI scanning was calculated. In baseline rs-fMRI, the mean head-motion values (x, y, z) were 0.16 ± 0.09, 0.38 ± 0.27, and 0.88 ± 0.63, and at follow-up, they were 0.17 ± 0.11, 0.34 ± 0.15, and 0.72 ± 0.48. Derivative or root mean square variance over voxels (DVARS) were also calculated, quantifying the mean change in image intensity between timepoints (Power et al., 2012). Time-series were then extracted from white matter and cerebrospinal fluid from preprocessed time-series data. Motion-regressors, DVARS, and white matter and cerebrospinal fluid time-series were included in a confounder matrix.

### Caudate-Related Connectivity Maps

**FIGURE 2:**
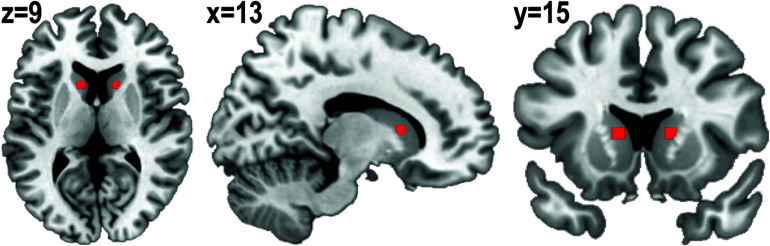
Binarized caudate seed. Three-millimeter spheres were placed centered on (x, y, z) = (13, 15, 9) and (x, y, z) = (–13, 15, 9). Right and left seeds were merged and regions outside of the anatomical caudate area were cut out.

Based on a previous report (Contreras-Rodríguez et al., 2017), 3-mm spheres were placed centered on (x, y, z) = (13, 15, 9) and (x, y, z) = (−13, 15, 9) as right and left caudate seeds. Right and left seeds were then merged and regions outside the anatomical caudate area were cut out and binarized to create the caudate seed (Figure 2). A time-series was extracted from unsmoothed preprocessed rs-fMRI data using the caudate seed. To create individual caudate connectivity maps, the extracted time-series from the seed was included in a general linear model using FSL’s fMRI Expert Analysis Tool. This model also included a confounder matrix as a nuisance covariate.

### Statistical Analysis

#### The Effect of Gender on BMI

We tested for gender differences in BMI at baseline and follow-up, and in the change in BMI, using a two-sample *t*-test.

#### Association Between Caudate-Related Connectivity and BMI

First, paired *t*-tests were conducted on individual connectivity maps derived from baseline and follow-up data to compare caudate-related FC.

Then, to assess the correlation between BMI and individual connectivity maps at baseline and follow-up, a group-level voxel-wise linear regression analysis with BMI as a covariate of interest was performed on individual caudate-related connectivity maps. To control for variance in age, age was included as a covariate of no-interest in the regression analysis. The cluster-forming threshold was set at *z* > 3.1. Clusters were then formed, associated *p* values were calculated, and *p* values that were > 0.05 (family wise error (FWE) corrected) were disregarded.

From brain regions in which there was a significant correlation between caudate-related FC and BMI at baseline, FC values (*z*-values) were extracted from connectivity maps at baseline and follow-up and compared. Further, correlation coefficients between extracted FC values and BMI were compared at baseline and follow-up using the “cocor” package version 1.1-3 in R (Diedenhofen and Musch, 2015).

#### Association Between Change in BMI and Change in Caudate-Related Connectivity

Changes in extracted FC values between baseline and follow-up (follow-up > baseline) were calculated, and the correlations between changes in extracted FC values and change in BMI (follow-up > baseline) were calculated using the Robust Correlation Toolbox in MATLAB R2020a (MathWorks, Inc., Natick, MA, United States) (Pernet et al., 2012). Robust correlation methods could provide better estimates of true associations, with accurate false-positive control and without loss of power by removing or down-weighting outliers and accounting for them in significance testing. To calculate skipped Pearson’s correlational coefficient, first the robust center of the data cloud was estimated using the minimum covariance determinant. Outliers were then identified using a projection technique. Lastly, Pearson’s correlation and associated *t* values were calculated using the remaining data. For correlational coefficients (*r*), 95% confidence intervals (CI) were calculated based on 1000 samples with the percentile bootstrap method implemented in the toolbox.

## Results

### Change in BMI and the Effect of Gender on BMI

The mean change in BMI was 0.25 ± 2.53 (range −4.33 to 6.53), and there was no significant difference in the change in BMI between male and female participants (*p* = 0.74, *t* = −0.34). The change in BMI was not significantly associated with the change in age (*p* = 0.09, *r* = 0.39).

There was no significant gender difference in BMI at baseline (*p* = 0.81, *t* = −0.24) or at follow-up (*p* = 0.36, *t* = −0.95).

### Internal State at the Rs-fMRI Sessions

The mean hunger rating at baseline was 4.6 ± 1.2, and at the follow-up timepoint, it was 4.1 ± 1.3. The mean fullness rating at baseline was 4.2 ± 1.2, and at the follow-up timepoint, it was 4.8 ± 1.2. Ratings for hunger and fullness at baseline did not differ significantly from the corresponding ratings at follow-up (*p* = 0.25, *t* = 1.18 for hunger and *p* = 0.17, *t* = −1.43 for fullness). There were no significant differences between ratings for hunger and fullness at baseline (*p* = 0.38, *t* = 0.89) or follow-up (*p* = 0.15, *t* = −1.51), suggesting that participants were neither hungry nor full at either of their rs-fMRI sessions.

### Association Between Caudate-Related Connectivity and BMI

There was no significant difference in caudate-related connectivity maps between baseline and follow-up.

**FIGURE 3:**
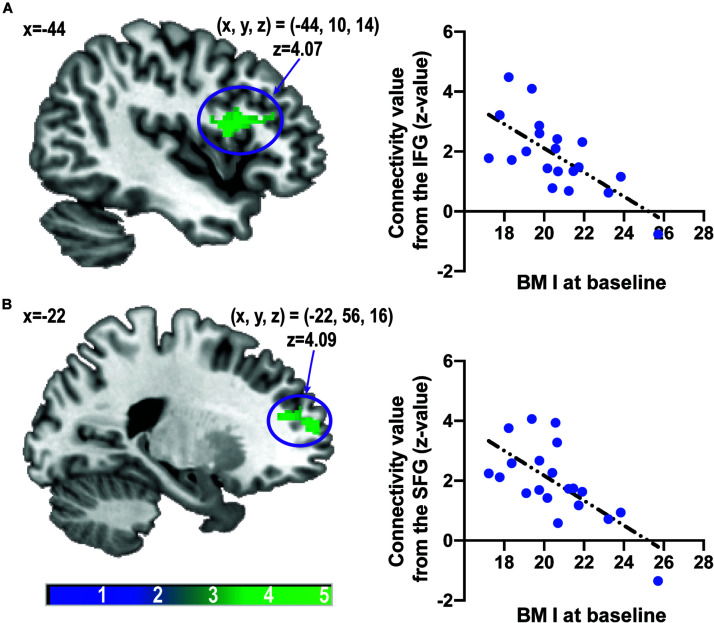
Associations between caudate-related functional connectivity and body mass index at baseline. Scatter plots depict associations between body mass index and caudate-related functional connectivity in the inferior frontal gyrus **(A)** and superior frontal gyrus **(B)**. The color bar depicts the *z*-value. IFG, inferior frontal gyrus; SFG, superior frontal gyrus.

Body mass index was inversely correlated with caudate-related FC in the inferior frontal gyrus (IFG), the opercular part [(x, y, z) = (−44, 10, 14), *z* = 4.07, P_FWE–corrected_ = 0.0014, cluster size = 187 voxels], and the superior frontal gyrus (SFG) [(x, y, z) = (−22, 56, 16), *z* = 4.09, P_FWE–corrected_ = 0.0048, cluster size = 154 voxels] (Figure 3). After controlling for age, the correlation between BMI and caudate-related FC in the IFG and SFG still remained significant [IFG: (x, y, z) = (−44, 10, 14), *z* = 3.94, P_FWE–corrected_ = 0.0089, cluster size = 137 voxels, SFG: (x, y, z) = (−22, 56, 16), *z* = 4.09, P_FWE–corrected_ = 0.0030, cluster size = 165 voxels].

There was no significant positive nor negative correlation between caudate-related FC and BMI at follow-up.

The linear relationship between caudate-related connectivity maps and BMI did not differ between baseline and follow-up.

Extracted caudate-related FCs in the IFG or SFG at baseline did not differ from those at follow-up (*p* = 0.61, *t* = −0.94 for FC in the IFG and *p* = 0.34, *t* = −0.97 for FC in the SFG).

Correlations between extracted FCs in the IFG or SFG and BMI at baseline were greater than those at follow-up (*p* = 0.030, *z* = −2.17 for the IFG and *p* = 0.039, *z* = −2.07 for the SFG).

Time of intervals between baseline and follow-up rs-fMRI sessions were not significantly correlated with caudate-related FC in IFG (*p* = 0.18, *r* = 0.31) or SFG (*p* = 0.41, *r* = 0.20), but change in BMI (*p* = 0.047, *r* = 0.45).

### Association Between Change in BMI and Change in Caudate-Related Connectivity

**FIGURE 4:**
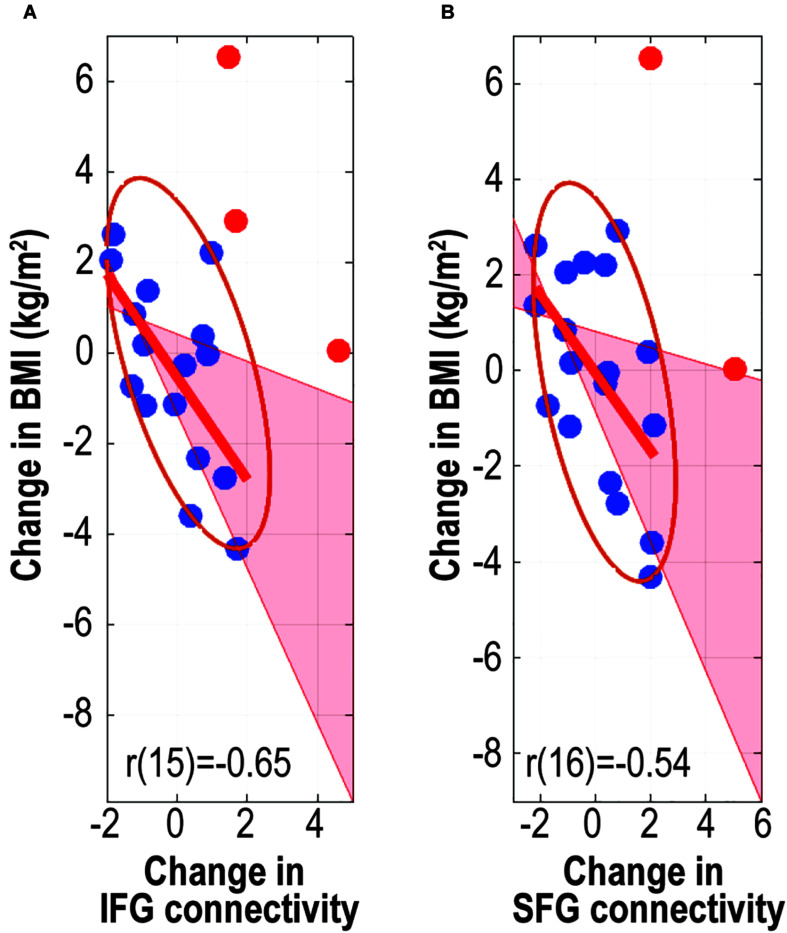
Associations between change in body mass index and change in caudate-related connectivity. Scatter plots depict associations between change in body mass index and caudate-related functional connectivity in the inferior frontal gyrus **(A)** and superior frontal gyrus **(B)**. *r* values are skipped Pearson’s correlational coefficients. Red data-points indicate outliers. IFG, inferior frontal gyrus; SFG, superior frontal gyrus.

There were significant negative correlations between the change in BMI and change in FC values (*z* values) from the IFG (skipped Pearson’s *r* [15] = −0.65, *p* = 0.002, 95% CI = [−0.90 to −0.19], number of outliers (NO) = 3) and from the SFG (skipped Pearson’s *r* [16] = −0.54, *p* = 0.015, 95% CI = [−0.80 to −0.10], NO = 2) (Figure 4). These results indicated that increased connectivity was correlated with reduced BMI gain.

### Associations Between Impulsivity and Age, Caudate-Related Connectivity, and BMI

Associations between each subscale of impulsivity and age at baseline and follow-up were tested. Increased attentional and motor subscales were positively correlated with age at baseline (*p* = 0.003, *r* = 0.63 for attentional subscale; *p* = 0.033, *r* = 0.48 for motor subscale) and increased attentional subscale with age at follow-up (*p* = 0.018, *r* = 0.52).

There was no significant difference in the correlation between age and attentional subscale between baseline and follow-up (*p* = 0.42, *z* = 0.81).

Associations between each subscale of impulsivity and caudate-prefrontal cortex connectivity in the IFG and SFG at baseline and follow-up were tested. Motor subscale was negatively correlated with FC in the IFG (*p* = 0.047, *r* = −0.45) at baseline and in the SFG at follow-up (*p* = 0.032, *r* = −0.48). Change in motor subscale was negatively associated with FC in the SFG (*p* = 0.030, *r* = −0.49).

Associations between each subscale of impulsivity and BMI at baseline and follow-up were tested. A significant association was not observed (*p* > 0.07 for all).

After the Bonferroni adjustment was performed for a multiple-comparison correction, the positive correlation between attentional impulsivity and age at baseline remained significant (significant level: *p* < 0.0125).

There was no significant gender difference in any subscales (*p* > 0.12 for all).

## Discussion

In the current study, greater caudate-IFG and caudate-SFG FCs were associated with lower BMI at baseline, and increased FC was negatively correlated with BMI gain at follow-up. Further, increased caudate-prefrontal cortex FC was associated with decreased impulsivity. To the best of our knowledge, the current longitudinal study is the first to show the association among the change in caudate-prefrontal cortex FC, BMI, and impulsivity in adolescents. The IFG, SFG, and caudate are involved in inhibitory control in adults (Guo et al., 2018; Cai et al., 2019) and children (Cai et al., 2019). Alterations in the IFG and SFG are involved in obesity, and gray matter reduction in the IFG has been reported in people with obesity (Herrmann et al., 2019). In adolescents, reduced cortical thickness in the IFG and SFG is reportedly associated with increased BMI and reduced cognitive function (Ronan et al., 2020). The IFG and SFG are involved in cognitive appetite control in adults (Tuulari et al., 2015) and attenuated caudate activation was observed during appetite control in middle aged people with obesity (Tuulari et al., 2015). Moreover, lower caudate responses to palatable liquid consumption in people with increased BMI are associated with elevated impulsivity (Babbs et al., 2013). Both caudate-IFG and caudate-SFG FC may be involved in cognitive appetite control and increases in these FCs may have preventive effects on excessive weight gain in adolescents.

In the present study, caudate-related FC at follow-up was not significantly associated with BMI, and the correlations between BMI and caudate-related FC in the prefrontal cortex were attenuated at follow-up. In young adolescents with healthy weight, increased impulsivity is associated with increased local FC in the caudate and decreased FC between the prefrontal cortex and caudate (Davis et al., 2013). Further, decreased caudate response to the inhibitory task was associate with increased impulsivity (Oliva et al., 2019). Given that age was positively associated with impulsivity at baseline in the current study, and the immature or obstruct PFC is involved with weight gain as well as elevated impulsivity (Lowe et al., 2019), attenuated FC between caudate-prefrontal cortex could be linked to increased impulsivity and lead to gained BMI at baseline. However, as the results showing that the associations between age and subscales of impulsivity at baseline differed from those at follow-up, the balance between sensation-seeking activation in the striatum and inhibitory control in the prefrontal cortex changes during adolescence (Romer, 2010), and this biological change may alter associations between caudate-related FC and BMI at follow-up compared to that at baseline. To examine this hypothesis, it is necessary to perform further follow-up observations.

Contrary to the study hypothesis, BMI was not significantly associated with caudate-related FC in reward regions, such as the striatum, midbrain, amygdala, insula, and orbitofrontal cortex (García-García et al., 2014; Stice and Yokum, 2016). Reward regions are related to weight gain and obesity (Stice and Yokum, 2016) and people with obesity reportedly have different caudate-related FC in reward-related regions (Lips et al., 2014). Therefore, associations between caudate-related FC in reward regions and BMI or BMI change may have been non-significant in the present study because almost all of the participants were of healthy weight.

The current study had several limitations. One is that it did not include obese adolescents. Compared with healthy weight adolescents, obese adolescents showed a greater caudate response to high calorie food images (Jastreboff et al., 2014), and a decreased caudate response following consumption of a milkshake (Stice et al., 2008b); therefore, caudate functional connectivity in adolescents with obesity is predicted to be different from that in healthy weight adolescents. While further investigation is needed to test this hypothesis, the current study provides insights into neural involvement in weight gain in adolescents. Second, not all participants’ BMI were measured on the day of the rs-fMRI session; however, all participants’ BMI were measured within 1 month before or after each rs-fMRI session. We assumed that their BMI did not change significantly within 1 month from the day of the rs-fMRI session because their self-reported BMI on the day of the rs-fMRI session were not significantly different from the measured BMI. However, since self-reported BMI is not reliable, small differences between the actual BMI on the day of the rs-fMRI session and that measured within a month from the day of the rs-fMRI session could potentially affect the association between BMI and caudate FC. Third, there was variation in the intervals between baseline and follow-up rs-fMRI sessions (mean 12.95 ± 3.02 months, range 11–22 months). Since there was no significant association between time of intervals and change in caudate FCs, this variation in the interval time appeared to have little effect on our analysis. However, as variance in the interval time could affect the correlation between the change in BMI and caudate FC, further studies should be conducted with fixed follow-up intervals. The fourth is that our sample size is not large. Although a preferable sample size for rs-fMRI studies has not been addressed in the literature to date, for a task-based fMRI experiment, Thirion et al. (2007) showed that a minimum sample size of 20 was necessary to ensure acceptable reliability. In addition, our sample is relatively homogeneous because all participants are Asian adolescents from similar residential areas, and we controlled the internal state for each participant. In addition, the effect size of correlation analysis between the connectivity values and BMI at baseline was calculated using G^∗^Power (Faul et al., 2007). All effect sizes were greater than 0.8. Therefore, the current results would be acceptable. However, it is preferable to perform further studies with greater sample size. Fifth, the results of the study do not demonstrate that there is a causal relationship between change in BMI and change in FC. Given that BMI gain is predicted by altered caudate function in adolescents, however (Stice et al., 2015), disrupted caudate-related FC may cause excessively increased BMI. Additional longitudinal studies are needed to investigate associations between development and the neuropathology of aberrant ingestive behavior in adolescents.

In conclusion, caudate-related FC in the inhibitory control regions has a preventive effect on excessive weight gain in adolescents. In the present longitudinal study, increased caudate-related FC in the prefrontal cortex – the inhibitory control region – was associated with lower BMI and impulsivity at baseline, and strengthened FC was inversely correlated with BMI gain and increment of impulsivity at follow-up in adolescents. These results suggest that developing inhibitory control may lead to successful prevention of childhood obesity.

## Data Availability Statement

The original contributions presented in the study are included in the article/supplementary material, further inquiries can be directed to the corresponding author.

## Ethics Statement

The studies involving human participants were reviewed and approved by the Ethics Committee of the Graduate School of Arts and Sciences at the University of Tokyo (approval number 513-2). Written informed consent to participate in this study was provided by the participants’ legal guardian/next of kin.

## Author Contributions

YN and SK: conceptualization and design, reviewing and editing the manuscript. YN and SO: data acquisition. YN: data analysis and writing the manuscript. All authors contributed to the article and approved the submitted version.

## Conflict of Interest

The authors declare that the research was conducted in the absence of any commercial or financial relationships that could be construed as a potential conflict of interest.
